# Fear of Predators Suppresses Neurogenesis in the Brains of Wild Songbirds

**DOI:** 10.1093/iob/obaf037

**Published:** 2025-10-21

**Authors:** L E Witterick, S Davidge, E C Hobbs, S A MacDougall-Shackleton, M Clinchy, L Y Zanette

**Affiliations:** Department of Biology, Western University, London, Ontario N6A 5B7, Canada; Advanced Facility for Avian Research, Western University, London, Ontario N6G 1G9, Canada; Department of Biology, Western University, London, Ontario N6A 5B7, Canada; Advanced Facility for Avian Research, Western University, London, Ontario N6G 1G9, Canada; Department of Biology, Western University, London, Ontario N6A 5B7, Canada; Advanced Facility for Avian Research, Western University, London, Ontario N6G 1G9, Canada; Advanced Facility for Avian Research, Western University, London, Ontario N6G 1G9, Canada; Department of Psychology, Western University, London, Ontario N6A 5C2, Canada; Department of Biology, Western University, London, Ontario N6A 5B7, Canada; Department of Biology, Western University, London, Ontario N6A 5B7, Canada; Advanced Facility for Avian Research, Western University, London, Ontario N6G 1G9, Canada

## Abstract

Fear of predation can lead to behavioral changes indicative of an enduring memory of fear, as acknowledged by both ecologists and biomedical scientists studying post-traumatic stress disorder (PTSD). Enduring memories are often linked to suppressed neurogenesis in laboratory rodents as a potential mechanism to prevent the replacement of existing memories. We used predator vocalizations to assess the enduring effects of fear on neurogenesis in a wild songbird, black-capped chickadees (*Poecile atricapillus*), quantifying cell proliferation (PCNA immunoreactivity), and immature neurons (doublecortin immunoreactivity) in both sexes. Seven days after predator cue exposure, we found suppression of hippocampal cell proliferation in males, with no effect in females, and suppression of immature neurons in the avian amygdala (medial ventral arcopallium) in both sexes. Our results are consistent with the hypothesis that animals retained an enduring memory of fear, with potential sex differences in the behavioral and ecological consequences of these enduring neuronal changes. Finding effects indicative of an enduring memory of fear in wild caught animals supports the notion that there may be evolutionarily adaptive value to retaining an enduring, PTSD-like memory.

## Introduction

Fear of predation is a common and widespread experience of wildlife, and can cause enduring effects on prey long after the immediate threat has passed, wherein memories of the life-threatening, traumatic experience of surviving a predator encounter persist (Zanette and Clinchy 2020). An enduring memory of past predator encounters fundamentally underlies the *Ecology of Fear* (Zanette and Clinchy 2019). Here, ecologists look beyond the immediate and transient stress response and focus on understanding how increased vigilance and reduced foraging of frightened animals can lead to lasting behavioral changes with population and community level consequences (Brown et al. 1999; Zanette and Clinchy 2019, 2020; Brown 2019; Gaynor et al. 2021). Little is known about the enduring effects of predator-induced fear on the brains of wildlife. A handful of observational studies, all focusing on fish, have demonstrated that living in areas with naturally high predation risk is correlated with alterations to brain size, cell proliferation, and hormone expression compared to those living with low predator pressure (Dunlap et al. 2016, 2019; Kotrschal et al. 2017; Reddon et al. 2018, 2022; Reyes et al. 2022). Only one previous study has experimentally demonstrated an enduring effect of fear on the brain in wild caught animals, with increased activation in brain regions associated with fear processing found in songbirds (Zanette et al. 2019). While it is implied that enduring effects in terrestrial wildlife are due to prey retaining a memory of predator-induced fear, the effects on the brain indicative of an enduring memory of fear have never been explicitly experimentally tested (Zanette and Clinchy 2017). There is, however, a large body of literature examining enduring fear effects in lab rodents where live predators or their cues (such as calls, mounts, or odors) are used to study post-traumatic stress disorder (PTSD; Deslauriers et al. 2018).

Predator stress, induced by presenting either a predator or their cues, is a commonly used technique for inducing PTSD-like symptoms in animal models due partly to the robust behavioral phenotypes produced and partly because such phenotypes can be produced after a single stressful experience, providing a valuable tool to understand the neural mechanisms behind the persistent and intrusive memories remaining after a life threatening event (Deslauriers et al. 2018). To distinguish PTSD-like effects from an immediate, transient anti-predator response, PTSD animal models also indicate that effects must endure for at least 7 days in a small bodied animal, such as rodents or songbirds (Deslauriers et al. 2018). The existing PTSD animal model literature focuses heavily on male animals, raising many questions about the enduring effects of predator-induced fear on female brains (Deslauriers et al. 2018). This is especially relevant because females may be more susceptible to predator-induced fear than males, at least in rodents (Deslauriers et al. 2018). Sex differences in selection pressures may incur additional fitness costs (Connallon et al. 2010). Moreover, understanding how both sexes respond to predator-induced fear is also ecologically important as it is the survival and fecundity of females that affects reproductive rates and therefore population growth. Assessing neural PTSD-like effects in both sexes and in a wild animal are necessary next steps in addressing concerns over whether the observed effects in the neurobiology of lab animal models can be generalized to their wild counterparts (Clinchy et al. 2011; Cohen et al. 2023).

While an enduring and disruptive memory of a traumatic event is a known symptom of PTSD, an enduring memory of how to anticipate and survive life threatening situations in a dynamic and changing environment would provide a fitness advantage for wildlife (Cohen et al. 2023). Behavioral changes in response to predators imply that prey animals are able to retain a memory of previous predator encounters as they alter their behavior to avoid future predator encounters (Brown et al. 1999; Zanette and Clinchy 2020). Experimental evidence in the laboratory indicates that the ability to retain fear memories alters anti-predator behavioral responses, which increase the likelihood of surviving future predator encounters (Crook et al. 2014; Zhong et al. 2023). This enduring effect of predator-induced fear may be most clearly seen in the concept of the *landscape of fear*, wherein the spatial variation in predation risk leads to alterations in prey movement across a landscape (Laundré et al. 2001, 2010; Gaynor et al. 2019). These proactive (Creel 2018) responses imply that animals are able to retain a memory of where they previously encountered predators in addition to the types of habitat where they might expect to encounter predators again (Suraci et al. 2019; Clermont et al. 2021; Epperly et al. 2021). Similarly, wildlife are well known to mount reactive anti-predator behavioral responses once an encounter with a predator has begun (Creel 2018), implying that prey retain a memory of their predators. If wild animals are forming enduring memories of predator experiences, we would expect to see an enduring signature in the brain indicative of memory retention.

If animals retain an enduring memory of fear, we might expect to see alterations to adult neurogenesis. The suppression of neurogenesis in the hippocampus promotes memory persistence, likely by preventing the replacement of existing memories (Frankland et al. 2013; Akers et al. 2014). For example, increasing neurogenesis through exercise has been shown to weaken the memory of a trauma and can reduce PTSD-like behaviors in lab mice (Fujikawa et al. 2024). Further, in mice displaying elevated anxiety-like behaviors in response to chronic stress, suppressed neurogenesis impaired recovery (Schoenfeld et al. 2019), implying that suppressed neurogenesis would indicate an enduring, PTSD-like memory of fear. Similarly, increased neurogenesis has been shown to weaken existing spatial memories (Epp et al. 2016), suggesting that suppressed neurogenesis may play an important role in maintaining the mental map that wildlife need to proactively (*sensu*  Creel 2018) respond to variations across the landscape of fear. We have previously demonstrated that fear of predators induced enduring effects on activation and hypervigilance behavior in wild-caught birds lasting at least 1 week after hearing predator vocalizations, which is consistent with an enduring memory of predator fear (Zanette et al. 2019). Therefore, we would expect to see a corresponding suppression in neurogenesis in birds under the same experimental conditions.

To experimentally test if predator-induced fear causes enduring changes in neurogenesis in wild animals, we followed methodology from both animal models for PTSD and the Ecology of Fear (Zanette and Clinchy 2019, 2020). We measured 2 stages of neurogenesis, quantifying both cell proliferation using proliferating cell nuclear antigen (PCNA), and immature neurons using doublecortin (DCX; von Bohlen und Halbach 2011). Following research using animal models for PTSD, we looked for effects enduring at least 7 days, which is considered an enduring effect in a small bodied animal (Deslauriers et al. 2018), and in 3 brain regions (the avian counterparts to the mammalian hippocampus, amygdala, and prefrontal cortex) most implicated in fear processing and PTSD (Diamond and Zoladz 2016; Akiki et al. 2017; Deslauriers et al. 2018; Lambert and McLaughlin 2019; Bauer 2023). To maximize the ecological relevance, we measured the responses of a songbird species (black-capped chickadee, *Poecile atricapillus;* hereafter “chickadees”). Chickadees were exposed to the vocalizations of their natural predators (or non-predators) and were then housed in naturalistic flocks for 7 days post-exposure. Several previous field manipulations using predator vocalizations have established that the fear of predators has population-level consequences in wild birds (Zanette et al. 2011; Dudeck et al. 2018; Allen et al. 2022). In the present study, we followed the same protocol which we previously used to demonstrate enduring, PTSD-like effects on neural activation and hypervigilance behavior in this species (Zanette et al. 2019). Our previously published study (Zanette et al. 2019) quantified the effects of predator cue exposure on: short-term neural responses, enduring neural responses, and behavioral responses. In the short term, chickadees showed elevated c-fos immunoreactivity in the avian counterparts to the hippocampus and amygdala in response to predator calls and conspecific alarm cue. One week after hearing predator calls, chickadees spent more time immobile in response to a conspecific alarm cue and presented elevated ΔFosB immunoreactivity in the avian counterparts to the hippocampus and amygdala. Here, we build upon these previous experiments to explicitly examine whether the enduring effects of fear of predators alters neurogenesis, because of the implications neurogenesis has for memory.

## Materials and methods

### Study species, animal housing, and predation risk manipulation

We used black-capped chickadees due to their abundance and year-round residency in Southern Ontario, and their ease in adapting to captivity. These same birds were used in a prior study to quantify the enduring effect of predator fear on ΔFosB in the brain (Zanette et al. 2019; see Discussion). Between September and November, 12 adult chickadees were captured using seed-baited Potter traps from sites around Western University, London, Ontario, Canada. Upon capture, chickadees were weighed, sex was estimated based on wing length (later confirmed with post-mortem examination of gonads), and chickadees were given a unique combination of color bands for individual identification. Chickadees were captured at least 7 days prior to the start of manipulations to acclimate to captivity and were housed in mixed sex groups in outdoor aviaries with ad libitum access to Mazuri small bird diet, black oil sunflower seeds, mealworms, and water.

Twelve Chickadees were randomly assigned to either the predator or non-predator control treatment, while we endeavored to maintain a balanced sex ratio between treatments (Predator: 2F, 4M; Non-Predator: 4F, 2M). These were the same 12 individuals in which we previously reported significant enduring effects of predator exposure on ΔFosB in the Hp and AMV. Individuals were relocated to cages within sound attenuating acoustic chambers 24 h prior to the manipulation. The chambers operated on a natural light cycle (11.5 h light: 12.5 h dark) with ad libitum access to food and water. Each chamber was outfitted with an MP3 player, speakers, and webcam for monitoring, and arranged so that the MP3 player and webcam could be operated without opening the chamber. Seven species were used in each treatment with species known to prey on chickadees selected for the predator treatment (Table 1), matched to non-predator species for sound characteristics between groups (Zanette et al. 2019). All species selected for the playbacks occur locally and their vocalizations would all be heard naturally by chickadees in the area. All calls were obtained from the Macaulay Library Database (Cornell University Lab of Ornithology, Ithaca, NY, USA) and the Xeno-Canto foundation (www.xeno-canto.org). Calls were broadcast at 74 dB SPL measured at the centre of the cage, with 5 min of calls playing at randomly selected intervals every hour. Our manipulation ran for 48 h, with playbacks broadcast 12 h per day during daylight hours. Following playbacks individuals were returned to their flocks in the outdoor aviaries.

**Table 1:** List of species used in the auditory playbacks for the chickadees.

Predators	Non-predators
Cooper’s hawk, *(Accipiter cooperii)*	Song sparrow *(Melospiza melodia)*
American crow *(Corvus brachyrhynchos)*	Mallard *(Anas platyrhynchos)*
Red-tailed hawk *(Buteo jamaicensis)*	Blue jay *(Cyanocitta cristata)*
Barred owl *(Strix varia)*	Northern leopard frog *(Lithobates pipiens)*
Sharp-shinned hawk *(Accipiter striatus)*	Hairy woodpecker *(Picoides villosus)*
Northern saw-whet owl *(Aegolius acadicus)*	Wood frog *(Lithobates sylvaticus*)
Merlin *(Falco columbarius)*	Downy woodpecker *(Picoides pubescens)*

*Note*: Predator and non-predator species were matched based on their acoustic call characteristics (frequency and maximum amplitude).

### Brain processing, immunohistochemistry, and image analysis

Seven days following the playback chickadees were euthanized by overdose of isoflurane, then transcardially perfused with 0.1M phosphate buffered saline (PBS; pH 7.4) and 4% paraformaldehyde. Brains were removed and placed in paraformaldehyde for 24 h, followed by 30% sucrose for 24 h until saturated and frozen at −80°C. Brains were sectioned to 40 μm coronal slices on a cryostat, taking 4 series starting from the end of the anterior commissure until the end of the cerebral lobes. Slices were stored in cryoprotectant at −20°C until immunohistochemistry began. We carried out immunohistochemistry on free-floating sections to label doublecortin (DCX [C-18] goat IgG, sc-8066, Santa Cruz Biotechnology) and proliferating cell nuclear antigen (PCNA [PC10] mouse IgG, sc-56, Santa Cruz Biotechnology) following established protocols (Diez et al. 2021), with the primary antibodies at a concentration of 1:250 and 1:1000 in 0.3% phosphate-buffered saline with triton (PBS/T), respectively. Sections were then labeled with a secondary antibody (horse anti-goat at 1:400 for DCX and goat anti-mouse at 1:250 for PCNA, Vector Laboratories) and visualized with diaminobenzidine solution.

**Fig. 1:**
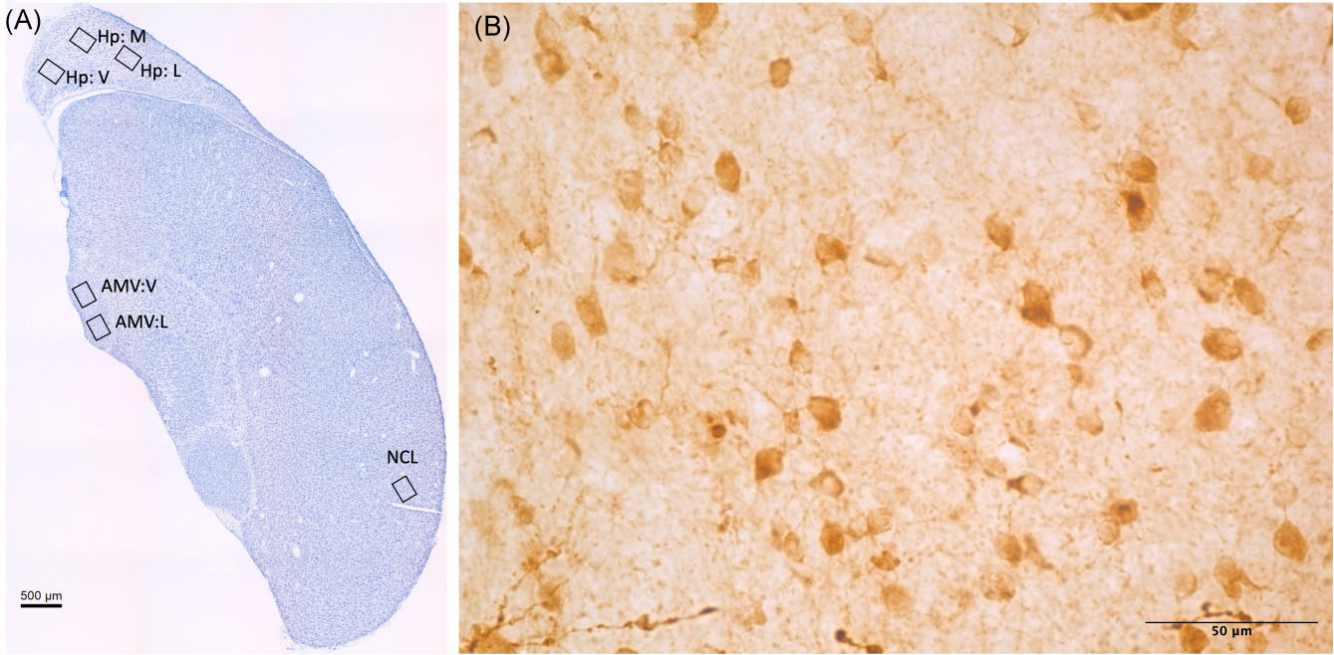
(A) Photomicrograph of a Nissl stained coronal section of chickadee telencephalon. Boxes depict locations where images were taken for DCX quantification within each brain region measured (L: lateral, M: medial, V: ventral). (B) Higher magnification photomicrograph of DCX immunoreactivity in the AMV.

**Fig. 2:**
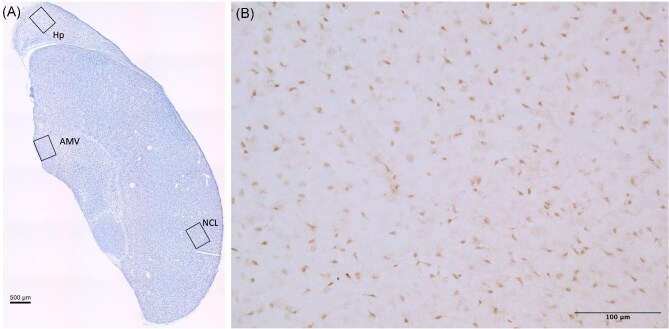
(A) Photomicrograph of a Nissl stained coronal section of chickadee telencephalon. Boxes depict locations where images were taken for PCNA quantification. (B) Higher magnification photomicrograph of PCNA immunoreactivity taken in the Hp.

For each marker, we quantified reactivity in the hippocampus (Hp; avian homologue to the mammalian hippocampus; Colombo and Broadbent 2000) medial ventral arcopallium (AMV; avian homologue to the amygdala; Yamamoto et al. 2005; Mello et al. 2019), and the caudolateral nidopallium (NCL; avian analogue to the mammalian prefrontal cortex; Herold et al. 2011). For DCX all images were taken using a 40× objective lens to capture z-stack images (Fig. 1). For the Hp, we took photos of the lateral, medial, and ventral subregions (Fig. 1). For the AMV, we took photos of the lateral and ventral subregions (Fig. 1). Within each of the Hp, AMV, and NCL, we took photos of each area of interest from each hemisphere on 5 slices. Images for the Hp were taken for the first 5 slices with both hemispheres present posterior to the anterior commissure. Images for the AMV and NCL were taken on slices where the region is present on both hemispheres starting from the anterior end of the AMV. We converted the images from color to 16-bit grayscale, enhanced the contrast, then used the thresholding tool in ImageJ (Schneider et al. 2012) to convert the DCX positive cells and fibres to black against a white background, manually adjusted threshold to best match cells, and quantified the percent cover of DCX immunoreactivity. The average percent cover was then calculated from all images quantified to have one data point from each brain region within each individual animal for analysis. The distribution of PCNA immunoreactive cells was similar to that reported in other studies (e.g., Diez et al. 2021). As expected, there was dense immunoreactivity along the ventricle walls where cell division occurs, but PCNA immunoreactivity was also found throughout the telencephalon (Fig. 2). For PCNA, all images were taken using a 20× objective lens, with one photo taken from each hemisphere on each slice that the region of interest was present (Fig. 2). We then calibrated ImageJ (Schneider et al. 2012) to the image measurement, and measured the area of interest in mm^2^. We converted the images from color to 16-bit grayscale, subtracted the background, and enhanced the contrast. We then used the thresholding tool to convert the PCNA positive nuclei to black cells on a white background, then used the count function to quantify the number of cells. Density was calculated as the PCNA positive cells/mm^2^ for each photograph. All images were collected and quantified without knowledge of the treatment groups or sex to avoid bias in the results.

### Statistical analysis

For each marker (i.e., DCX and PCNA) the mean value was calculated for each brain region within each individual animal. A two-way Analysis of Variance (ANOVA) was used to analyze the effects on each marker within each brain region measured, with treatment and sex as the independent variables and the percent cover of DCX-immunoreactive cells or the density of PCNA-immunoreactive cells as the dependent variable. A Dunnett’s post hoc test was used for PCNA, which compared non-predator males against the predator males, predator females, and non-predator females. When working with wild animals, it is necessary to balance the need to maximize our sample size with the need to minimize the number of animals euthanized. To support the above parametric analyses and minimize the potential for error due to sample size, we conducted additional non-parametric tests to ensure that our results were robust. First, to corroborate the effect of treatment, we analyzed the effect of treatment with a non-parametric Mann–Whitney U test on *z*-scores of our data. The *z*-scores standardized the data according to sex to control for any variation due to sex in the response to the experimental treatments. To corroborate all two-way ANOVA results, we then followed up with a permutation tests which are frequently used in neuroscience (Nichols and Holmes 2007). A permutation test is a non-parametric estimate of the population distribution that is used to estimate how rare the observed values are by sampling all possible permutations of the data without replacement and is robust and effective for small sample sizes. The Freedman–Lane permutation method was used because it is the most accurate and powerful in controlling for type 1 errors (Winkler et al. 2014) and ran 10,000 permutations with the permuco package in R (following Frossard and Renaud 2021). All Mann–Whitney and permutation results supported the parametric ANOVA results and are outlined in Supplementary Material 1. We report results from our parametric ANOVAs in the main text. All analyses were conducted in RStudio Version 1.4.1103.

## Results

### Enduring effects of fear on cell proliferation—PCNA

**Fig. 3:**
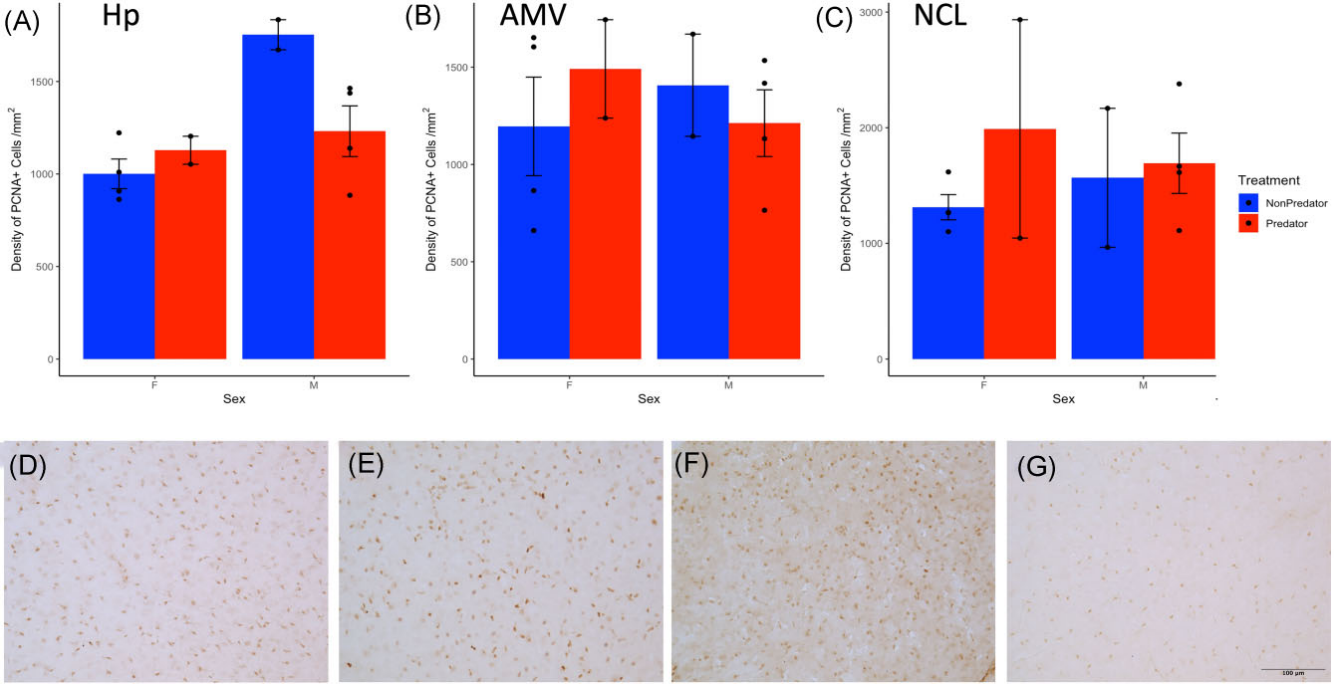
Cell proliferation: The effects of predator induced fear on the PCNA + cell density in the (A) hippocampus (Hp), (B) medial ventral arcopallium (AMV), and (C) the caudolateral nidopallium (NCL). Bars indicate means ± standard error, filled circles indicate individual data. Example photomicrographs of PCNA immunoreactivity in the Hp of a (D) non-predator female, (E) predator female, (F) non-predator male, and (G) predator male.

Exposure to predator playbacks 1 week earlier had an enduring effect on PCNA immunoreactivity in the hippocampus (Hp) in males but not females. (Fig. 3A; 2-way ANOVA treatment x sex interaction: *F*_1,8_ = 6.89, *P* = 0.03; main effect on sex: *F*_1,8_ = 18.49, *P* = 0.003; main effect of treatment: *F*_1,8_ = 0.53, *P* = 0.49). Males in the non-predator control treatment displayed 43% more PCNA-immunoreactive cells compared to their female counterparts (Dunnett’s post hoc, *P* = 0.007). In the predator treatment in contrast, males and females did not differ significantly in PCNA immunoreactive cells in the Hp, because 7 days after hearing predator playbacks, males demonstrated 30% lower PCNA immunoreactive cells relative to males in the control treatment (*P* = 0.045), whereas females exhibited no change compared to controls (Fig. 3A). There were no statistically significant main effects or interactions regarding PCNA immunoreactivity in the medial ventral arcopallium (AMV; Fig. 3B; all *P* > 0.3) or the caudolateral nidopallium (NCL; Fig. 3C; all *P* > 0.2)

### Enduring effects of fear on immature neurons—DCX

**Fig. 4:**
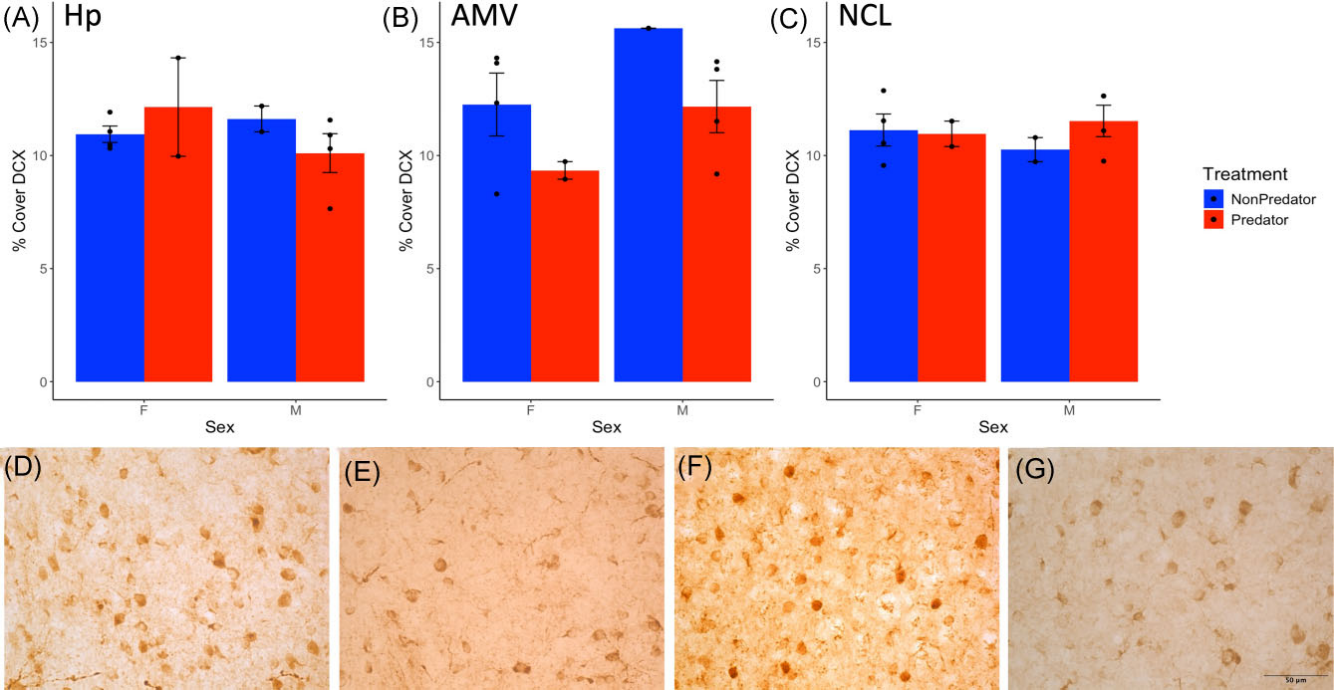
Immature neurons: The effects of predator-induced fear on the percent cover of DCX + cells in the (A) hippocampus (Hp), (B) medial ventral arcopallium (AMV), and (C) the caudolateral nidopallium (NCL). Bars indicate means ± standard error, filled circles indicate individual data. Example photomicrographs of DCX immunoreactivity in the AMV of a (D) non-predator female, (E) predator female, (F) non-predator male, and (G) predator male.

Predator playback exposure also had enduring effects on DCX immunoreactivity, but in this case, in the medial ventral arcopallium (AMV; the avian homologue to the amygdala) and irrespective of sex. Chickadees who heard predator calls 1 week earlier showed a 16% reduction in DCX immunoreactivity in the AMV (Fig. 4B; *F*_1,8_ = 5.48, *P* = 0.047). Sex did not have a statistically significant main effect (*F*_1,8_ = 5.18, *P* = 0.052; means ± SE, males versus females: 13.32 ± 1.03 versus 11.28 ± 1.08) and there was no treatment by sex interaction (*F*_1,8_ = 0.04, *P* = 0.85). We found no main effects or interaction of treatment and sex in the hippocampus (Hp; Fig. 4A; all *P* > 0.2) or the caudolateral nidopallium (NCL; Fig. 4C; all *P* > 0.3).

## Discussion

Our results demonstrate that a chronic perceived sense of heightened danger from predators leaves an enduring effect on neurogenesis. This is indicative of an enduring memory of fear, with some evidence of sex dependence, in brain regions associated with fear processing lasting at least 1 week after the threat has been removed. The assumption that the neural response measured is a fear response to the predator cues presented is corroborated by previous work using identical methodology to demonstrate enduring hypervigilance behavior in chickadees in response to a conspecific alarm cue 1 week after hearing predator calls (Zanette et al. 2019), along with multiple field experiments reporting behavioral and reproductive effects in free-living birds clearly demonstrative of fear responses to predator playbacks (Zanette et al. 2011; Hettena et al. 2014; Dudeck et al. 2018; Zanette and Clinchy 2020; Allen et al. 2022). In the AMV (a proposed homologue of the mammalian amygdala; Yamamoto et al. 2005; Mello et al. 2019), immature neurons (DCX) were significantly suppressed for both males and females. In the Hp, immature neurons did not vary with treatment, but we did find an effect of treatment on cell proliferation (PCNA) according to sex. Males significantly reduced cell proliferation 1 week after hearing predators, while females showed no change between treatments. We also found a sex difference in cell proliferation in the control treatment, suggesting that males and females may face different behavioral and ecological consequences from fear-induced alterations to hippocampal neurogenesis and highlighting the importance of including both sexes in any studies on neurobiology. Unlike the Hp and AMV, the NCL did not show an enduring effect of fear on either cell proliferation or immature neurons.

Our results revealed that exposure to predator vocalizations reduced hippocampal cell proliferation, defined here as PCNA-ir cells, in males, while females showed no statistically significant difference. PCNA has been widely used to label proliferation of new neurons detected throughout the avian brain (Mazengenya et al. 2017, 2018, 2020; Diez et al. 2021); however, it can also be found in non-neuronal dividing cells (Wang 2014). It is widely accepted that adult neurogenesis occurs in the hippocampus (Christie and Cameron 2006), with cell proliferation in the songbird brain beginning in the subventricular zone (Pytte 2016). The PCNA-ir cells measured in this experiment are likely very young cells that have very recently migrated away from the subventricular zone while PCNA is still present in the cell, and are comparable to other studies showing PCNA-ir cells in many regions throughout the avian brain (Mazengenya et al. 2017, 2018, 2020; Diez et al. 2021). PCNA has been demonstrated to have a 20 h half-life in rodents (Bravo and Macdonald-Bravo 1987). This has not been directly tested in birds, but if PCNA does show a comparable half-life, this would suggest both that fear is directly impacting the rate of hippocampal neurogenesis and that this effect endures long after the threat has passed. Indeed, this same pattern has been demonstrated in rats in the lab in which exposure to fox odor led to a decrease in hippocampal cell proliferation 24 h later in males with no effect in females (Falconer and Galea 2003). Similarly, a suppression in cell proliferation was also documented in the forebrain of fish living in a naturally high predator environment compared to a population experiencing naturally low predation pressure with no difference reported between sexes (Dunlap et al. 2016). Ours is the first experimental evidence that fear affects cell proliferation in brain regions associated with fear in wildlife and the first to demonstrate these effects in any bird.

Cell proliferation in the Hp also differed between the sexes in our non-predator control treatment, wherein males had 43% more PCNA immunoreactive cells than females, which is consistent with previously reported sex differences in the ventricular zone of the avian brain (Katz et al. 2008). In winter flocks, chickadees form stable dominance hierarchies with males generally dominant over females (Smith 1991). Dominant chickadees have been shown to have more newly proliferated cells in the ventricular layer of the Hp than do subordinates (Pravosudov and Omanska 2005), which may explain our results in the control group. If reduced cell proliferation enhances memory retention as previous studies have indicated (Frankland et al. 2013; Akers et al. 2014), then it is possible that by suppressing hippocampal cell proliferation in response to predators male chickadees make a trade-off that prioritizes memory of predators at the expense of maintaining dominance status. Dominant males and their female mates typically access the highest quality breeding sites during the following breeding season (Smith 1991). However, even a lower quality breeding site would still give a greater potential fitness benefit than being killed by a predator before the breeding season begins. Further behavioral studies would be needed to better elucidate the ecological consequences of our reported sex differences in hippocampal cell proliferation. Our results suggest that including both sexes may provide useful insight into how fear of predators operates, and that the historic trend of favoring males in lab animal models for PTSD (Deslauriers et al. 2018) could potentially be restricting our understanding of fear.

In the AMV (proposed homolog of the mammalian amygdala, Yamamoto et al. 2005; Mello et al. 2019), we found a reduction in immature (DCX+) neurons in both sexes 1 week after the predator treatment. While the AMV and its mammalian counterpart the amygdala are not historically known as key neurogenic regions, there is evidence for neurogenesis occurring in the mammalian amygdala (Fowler et al. 2008; Jiang et al. 2014; Jhaveri et al. 2018; Jurkowski et al. 2020; Sánchez-Gomar et al. 2024). In the avian brain, adult neurogenesis is widespread (Brenowitz and Larson 2015), suggesting that neurogenesis outside of the hippocampus is much more common in birds than mammals.

While quantifying DCX immunoreactivity with percent cover does include both the migrating fusiform cells and the recently differentiated multipolar cells (Balthazart and Ball 2014), fusiform cell counts have been shown to positively correlate to percent cover of DCX-ir cells in regions associated with avian vocal control and auditory perception (Aitken et al. 2024), suggesting that treatment effects on percent cover of DCX-ir cells capture differences in newly formed, migrating cells. Given that we found enduring effects in the AMV only on immature neurons (DCX), with no corresponding effect on cell proliferation (PCNA), it is also possible that these immature neurons migrated from the ventricular zone at a later stage of the cell cycle, because doublecortin expression can be measured in cells for at least 60 days from formation (Balthazart and Ball 2014; Vellema et al. 2014) and has been shown to correlate to neuronal migration (Hannan et al. 1999). Neuronal migration may also explain the lack of a statistically significant enduring effect of fear on immature neurons in the Hp, while males showed a significant reduction in hippocampal cell proliferation, particularly if predator induced fear plays a role in the rate at which these newly proliferated neurons are migrating from the Hp to other areas of the brain.

Our results suggest that exposure to predator cues suppressed neurogenesis, as indicated by both the immature neurons of the AMV of males and females and in cell proliferation in the Hp of males. Reduced hippocampal neurogenesis has been demonstrated to increase memory persistence (Akers et al. 2014), while increased neurogenesis weakened trauma related memories and attenuated PTSD-like behaviors in lab mice (Fujikawa et al. 2024). Reduced neurogenesis has also been shown to impede reference memory reversal in chickadees (Guitar and Sherry 2018), supporting the idea that suppression of neurogenesis helps prevent the replacement of existing memories (Frankland et al. 2013). While the Hp is most often associated with memory formation, particularly in the avian brain (Sherry and Hoshooley 2007), the amygdala has also been found to play an essential role in the consolidation, storage, and recall of “emotional” memories, particularly those memories provoked by fear (Richter-Levin 2004; Hermans et al. 2014; Diamond and Zoladz 2016; Hammack et al. 2023a, 2023b). Fos activation in the amygdala has also been used as a marker of remote memory recall (Hammack et al. 2023a, 2023b), and is consistent with the increased ΔFosB activation in response to predator exposure previously documented in the brains of the very same individuals assayed in this study (Zanette et al. 2019). Chickadees that were part of this experiment also demonstrated an enduring behavioral response suggestive of an enduring memory of fear, in the form of increased hypervigilance in response to a conspecific alarm cue 1 week after hearing predator cues (Zanette et al. 2019). Strongly corroborating our present results is that we also documented suppressed DCX immunoreactivity in the AMV 1 week after predator exposure, in a previous fear manipulation experiment we conducted on wild birds (brown-headed cowbirds) in semi-natural conditions in the field (Witterick et al. unpublished results). It is also possible that instead of being actively suppressed, the effects on neurogenesis were instead indirectly affected by fear as a result of differential exercise in response to our treatments; exercise being commonly used to induce neurogenesis in laboratory animal models (Epp et al. 2021). In this case, the enduring increase in hypervigilance behavior reported in the animals who heard the predator treatment would presumably have led them to be less active throughout the experimental period. Thus, the treatment effect observed could have resulted from less exercise in the predator treatment and more in the non-predator treatment. For humans with PTSD, it is a hyperfunctional amygdala that is thought to maximize individual survival through heightened vigilance to minimize the chance of a surprise predator encounter, an evolutionarily primitive mechanism to maximize survival (Diamond and Zoladz 2016). This suggests that the enduring suppression of immature neurons seen in the AMV is contributing to an enduring memory of predator-induced fear; thereby better readying the individual to avoid, or react to, the next predator encounter, and so have a better chance of survival.

Sex differences in rates of cell proliferation without corresponding sex effects on immature neurons have previously been attributed to differences in cell survival (Galea et al. 2006), which may be a factor in why we saw suppression of hippocampal cell proliferation only in males but suppression of immature neurons in the AMV in both sexes. While it is possible that the reported sex differences were influenced by the hormonal differences resulting from the differential sex ratios within our treatment groups, this is unlikely to have substantially impacted the results given that birds were in non-breeding condition so we would expect the influence of gonadal hormones to be minimal (Tamai and Yoshimura 2017; Shinomiya and Yoshimura 2018). While we did not detect a statistically significant main effect of sex on immature neurons, negative results should be interpreted with caution given the limited sample size and resulting power of the statistical analyses and could be an interesting direction for further investigation. Further research quantifying apoptosis along with neurogenesis in both sexes could also be an interesting next step to understanding how much of the reduction in immature neurons is caused by a reduction in new cells being produced compared to how many cells are undergo apoptosis before reaching maturity. Given that cell proliferation converges between the sexes in the predator treatment and there is no sex difference in the suppression of immature neurons, it is possible that the sex difference represents 2 different regulatory pathways to a hyperfunctional amygdala retaining an enduring memory of fear.

Enduring suppression of neurogenesis indicative of an enduring memory of fear, particularly in wild caught animals who likely already had a baseline level of previous predator experience, supports the notion that there may be an evolutionarily adaptive advantage to PTSD if the memory of that traumatic event is advantageous for survival (Cohen et al. 2023). Retaining an enduring memory of fear may also have evolutionary advantages enduring past the lifetime of the individual, as adult mice who experienced a 5 min predator encounter produced offspring and grand-offspring who showed enduring anxiety-like behavior and hyperarousal measured in response to mild stressors (2 min predator exposure), in addition to increased hippocampal activation in the offspring (Bhattacharya et al. 2023). Intergenerational transmission of PTSD has also been documented in humans, although further research is needed to elucidate the mechanisms behind this (reviewed in Bowers and Yehuda 2016; Yehuda and Lehrner 2018). To further understand these potential adaptive advantages, the next step would be to investigate whether comparable enduring effects can be seen in wild animals outside of the controlled laboratory environment, with predator exposure occurring either under semi-natural conditions in captivity or in free-living wildlife. Finding PTSD-like effects indicative of an enduring memory of fear in a wild caught animal suggests that while the costs of a predator encounter may be far greater than the opportunity costs of reduced foraging as seen in the ecology of fear (Brown et al. 1999; Brown 2019), these enduring effects of fear are likely common in nature and may give an evolutionarily adaptive advantage.

## Supplementary Material

obaf037_Supplemental_File

## Data Availability

The data underlying this article will be shared on reasonable request to the corresponding author.
