# Supplementary material for: Fear of Predators Suppresses Neurogenesis in the Brains of Wild Songbirds
Source: Integr Org Biol. 2025 Oct 21;7(1):obaf037. doi: 10.1093/iob/obaf037 (PMC12574329; doi:10.1093/iob/obaf037)
Supplement: obaf037_Supplemental_File [file obaf037_supplemental_file.docx]

Supplementary Material 1: Statistical results for the parametric ANOVA and the permutation ANOVA.

| Marker | Brain Region | Factor | Parametric F | Parametric p | Permuted p | Mann-Whitney |
| --- | --- | --- | --- | --- | --- | --- |
| PCNA | Hp | Treatment | 0.53 | 0.49 | 0.15 | 0.70 |
|  |  | Sex | 18.49 | 0.003 | 0.01 |  |
|  |  | Treatment*Sex | 6.89 | 0.030 | 0.026 |  |
|  | AMV | Treatment | 0.67 | 0.44 | 0.85 | 0.94 |
|  |  | Sex | 0.34 | 0.57 | 0.88 |  |
|  |  | Treatment*Sex | 0.92 | 0.36 | 0.37 |  |
|  | NCL | Treatment | 1.41 | 0.27 | 0.35 | 0.59 |
|  |  | Sex | 0.20 | 0.66 | 0.96 |  |
|  |  | Treatment*Sex | 0.47 | 0.51 | 0.52 |  |
| DCX | Hp | Treatment | 0.75 | 0.41 | 0.88 | 0.81 |
|  |  | Sex | 0.24 | 0.64 | 0.51 |  |
|  |  | Treatment*Sex | 1.91 | 0.20 | 0.21 |  |
|  | AMV | Treatment | 5.49 | 0.047 | 0.042 | 0.041 |
|  |  | Sex | 5.18 | 0.052 | 0.051 |  |
|  |  | Treatment*Sex | 0.04 | 0.85 | 0.87 |  |
|  | NCL | Treatment | 0.02 | 0.88 | 0.50 | 0.59 |
|  |  | Sex | 0.62 | 0.45 | 0.85 |  |
|  |  | Treatment*Sex | 0.85 | 0.38 | 0.37 |  |
